# Modeling and Simulation of Fabricated Graphene Nanoplates/Polystyrene Nanofibrous Membrane for DCMD

**DOI:** 10.3390/polym13172987

**Published:** 2021-09-03

**Authors:** Ahmad Abdullah, Abdulaziz Al-Qahatani, Mohammed Alquraish, Colin Bailey, Ahmed El-Shazly, Salah El-Mofty

**Affiliations:** 1 Department of Civil Engineering, College of Engineering, University of Bisha, P.O. Box 551, Bisha 67614, Saudi Arabia; selmofty@ub.edu.sa; 2 Department of Civil Engineering, Faculty of Engineering, Aswan University, Aswan 81542, Egypt; 3 Department of Biology, College of Science, University of Bisha, P.O. Box 551, Bisha 61922, Saudi Arabia; arabe@ub.edu.sa; 4 Department of Mechanical Engineering, College of Engineering, University of Bisha, P.O. Box 551, Bisha 67614, Saudi Arabia; malqraish@ub.edu.sa; 5 School of Engineering and Materials Science, Queen Mary University of London, Mile End Road, London E1 4NS, UK; principal@qmul.ac.uk; 6 Chemical and Petrochemicals Engineering Department, Egypt-Japan University of Science and Technology (E-JUST), Alexandria 21934, Egypt; elshazly_a@yahoo.com

**Keywords:** DCMD, SEM, composite membrane, graphene nanoplates, polystyrene, CFD

## Abstract

Membrane distillation is an active technique that provides pure water with very good rejection and could be applied to water of extremely high salinity. The low productivity of membrane distillation needs intensive efforts to be competitive with other desalination techniques. In this current study, a composite (PS/GNP) membrane, which is composed of polystyrene (PS) based and 0.25% weight percent graphene nanoplates (GNP) has been fabricated via electrospinning and compared with the blank PS membrane. SEM, FTIR, contact angle and porosity characterization have been performed, and the results show that the validity of the predefined conditions, and the contact angle of the composite membrane, which is found to be 91.68°, proved the hydrophobic nature of the composite membrane. A numerical simulation using Ansys 2020 software has been introduced to study the performance of the fabricated composite membrane when used in direct contact membrane distillation (DCMD). The numerical model has been validated with experimental work from the literature and showed an excellent match. The blank PS and composite PS/GNP membranes have been investigated and compared at different operating conditions, i.e., hot water supply temperature and system flow rate. The results show that the composite PS/GNP membrane outperforms the blank PS membrane at all studied operating conditions.

## 1. Introduction

Membrane distillation technique (MD) is based on a thermal concept for operation; the difference in temperature between the hot and cold streams promotes the vapor pressure difference, which drives the vapor to be transferred from the hot to cold side [1,2,3]. There are four common configurations for membrane distillation, which are Direct Contact membrane distillation (DCMD) where the feed and permeate are contacting both membrane surfaces directly, Air-Gap membrane Distillation (AGMD) where a condensation surface is used at the permeate side and is separated from the membrane surface by means of an air gap, and Vacuum membrane distillation (VMD) in which a vacuum is initiated at the permeate side to increase the amount of vapors transferred through the membrane and Sweeping Gas membrane Distillation (SGMD) [4,5]. However, the most popular is direct contact membrane distillation (DCMD) because it is simple and effective [6,7].

Various studies have been conducted before to improve the MD performance, which may deal with enhancing thermal performance [8], membrane hydrophobicity and characteristics [5], and improving life span via addressing fouling and scaling [1]. On the other hand, other studies were performed to improve its economics to be a competitor with other techniques via integrating the MD with solar energy to eliminate the external power supply [3,9].

For the first time, Niknejad et al. [10] produced a superhydrophobic membrane using polymethyl methacrylate (PMMA) fabricated via electro-blowing technique. This work showed a promising performance compared to commercially available membranes. A modified surface membrane has been introduced by Li et al. [11], which was proven to have an excellent hydrophobic property (i.e., 162° contact angle), outstanding wetting behavior and steady operation. They concluded that the modified surface membrane is applicable and has a promising performance when applied to DCMD.

The effect of feed inlet temperature, permeate inlet temperature, flow rate and salt concentration has been introduced numerically and validated experimentally by Park et al. [12]. They concluded that feed inlet temperature significantly affects the system productivity more than permeate temperature. They also investigated the effect of spacers on the system performance and concluded that such spacers enhanced the system performance via decreasing the temperature polarization. Furthermore, an experimentally validated model has been introduced to predict the response of the MD system to the operating conditions by Ismail et al. [13]. The authors proved the validity of their model and introduced a model to calculate the membrane thermal conductivity.

Temperature polarization occurring in the flow channels negatively affects the MD performance. A comprehensive review was conducted by Anvari et al. [14], which introduced the most recent advances to address temperature polarization in MD. They highlighted the turbulence promoters and membrane surfaces’ coating using thermally conductive material to reduce temperature polarization in MD and enhance the overall performance. Moreover, the effect of spacer on the DCMD performance besides operating conditions (feed temperature, velocity, and salt concentration) has been investigated experimentally by Ve et al. [15]. They concluded that the plastic spacer had the best performance in their study, and the flow rate was proven to be the most critical operating condition on the mass transfer through the membrane.

Due to the great importance of MD technology, researchers focused on the fabrication material and techniques to improve the membranes’ characteristics [16]. The authors also introduced the solution for fouling, which may be organic or inorganic. Furthermore, a comprehensive review of polymeric membranes has been conducted by Ravi et al. [17]. They introduced the fabrication and characterization of the polymeric membranes as its applicability on the MD technique compared to other membranes. In addition, an electrospun polystyrene membrane has been fabricated successfully [18]. The authors applied the fabricated membrane to DCMD for industrial water treatment, and the results show excellent operating performance and salt rejection.

A different weight percent of PS was examined to obtain the optimum weight percent, which was found to be 18% [19]. The authors concluded that feed inlet temperature, as well as the porosity, have a significant effect on the system productivity.

From the open literature, the MD technique deserves more and more improvements to be competitive with other techniques. Up to the authors’ best knowledge, the composite Polystyrene (PS) with Graphene nanoparticles (GNP) membrane is not investigated. Accordingly, the current work aims to introduce a new composite membrane which is composed of PS with GNP to obtain a new PS/GNP membrane. The fabricated membrane is characterized to confirm the successful fabrication, then the performance of the fabricated membrane is introduced using a DCMD unit. The proposed work has two original sides, the main side is the fabrication of the composite PS/GNP membrane and comparison with blank PS. The second point is the design of the numerical model which is suitable for predicting the performance of any fabricated membrane on the DCMD system.

## 2. Materials and Methods

Polystyrene (PS) pellets of 192000 molecular weight (purchased from Alpha Chemika, India), *N*, *N*-dimethyl formamide (DMF) (99.8% GC, ACS reagent, purchased from Sigma-Aldrich, Germany), and Graphene nanoplatelets (GNPs) (Carbon > 85 wt.%, purchased from Sigma-Aldrich, Germany) were used.

### 2.1. Fabrication Process

At first, the weight percent of PS was selected to be 18% to obtain the optimum performance [19]. The PS pellets were dissolved in DMF, and the solution was kept for 6 h on a stirrer at 27 °C to obtain a homogeneous solution. The polymer solution was prepared to be ready for electrospinning (Electrospinning/spray System, NanoNC, Seoul, Korea) under the following conditions:

1. Solution injection rate = 0.6 mL/h.
2. Tip to collector distance = 18 cm.
3. Potential difference = 28 kV.
4. Solution volume = 3.5 mL.

The weight percent (wt.%) of the GNP was changed during the current work to study the effect of the weight percent on the membrane performance. The fabricated composite membranes were composed of PS and 0.25 wt.% of GNP. The target wt.% of GNP was added to the polymer solution after dissolution and a 1 h stirring process, followed by a similar time sonication process. Then, the composite solution was introduced to the electrospinning to obtain the full matrix membrane, which was left overnight in the oven at 60 °C.

### 2.2. Characterization

The following characterizations have been introduced in the current work to highlight the characteristics of the composite fabricated membrane:

1. Scanning Electron Microscope (SEM).
2. Fourier-Transform Infra-Red (FTIR).
3. Contact Angle.
4. Porosity.

## 3. Mathematical Model

The MD system is restricted with the following governing equations:

### 3.1. Mass Conservation

(1) $$\frac{\partial \rho U_{x}}{\partial x}+\frac{\partial \rho U_{y}}{\partial y}+\frac{\partial \rho U_{z}}{\partial z}=0\;$$

### 3.2. Momentum Conservation

x-Direction:

(2) $$U_{x}\frac{\partial {\rho U}_{x}}{\partial x}+U_{y}\frac{\partial {\rho U}_{x}}{\partial y}+U_{z}\frac{\partial {\rho U}_{x}}{\partial z}=-\frac{\partial P}{\partial x}+\mu \left(\frac{\partial^{2}U_{x}}{\partial x^{2}}+\frac{\partial^{2}U_{x}}{\partial y^{2}}+\frac{\partial^{2}U_{x}}{\partial z^{2}}\right)$$

y-Direction:

(3) $$U_{x}\frac{\partial {\rho U}_{y}}{\partial x}+U_{y}\frac{\partial {\rho U}_{y}}{\partial y}+U_{z}\frac{\partial {\rho U}_{y}}{\partial z}=-\frac{\partial P}{\partial y}+\mu \left(\frac{\partial^{2}U_{y}}{\partial x^{2}}+\frac{\partial^{2}U_{y}}{\partial y^{2}}+\frac{\partial^{2}U_{y}}{\partial z^{2}}\right)$$

z-Direction:

(4) $$U_{x}\frac{\partial {\rho U}_{z}}{\partial x}+U_{y}\frac{\partial {\rho U}_{z}}{\partial y}+U_{z}\frac{\partial {\rho U}_{z}}{\partial z}=-\frac{\partial P}{\partial z}+\mu \left(\frac{\partial^{2}U_{z}}{\partial x^{2}}+\frac{\partial^{2}U_{z}}{\partial y^{2}}+\frac{\partial^{2}U_{z}}{\partial z^{2}}\right)$$

### 3.3. Energy Conservation

(5) $$U_{x}\frac{\partial {\rho C}_{p}T}{\partial x}+U_{y}\frac{\partial {\rho C}_{p}T}{\partial y}+U_{z}\frac{\partial {\rho C}_{p}T}{\partial z}=k\left(\frac{\partial^{2}T}{\partial x^{2}}+\frac{\partial^{2}T}{\partial y^{2}}+\frac{\partial^{2}T}{\partial z^{2}}\right)$$

where U*_x_*, U*_y_*, and U*_z_* are the three components of the velocity vector; P, T, and ρ are pressure, temperature and density, respectively.

Additionally, the targeted parameters in the current study are the system productivity (m), which is also named permeate flux, the overall efficiency of the system (η), and the coefficient of temperature polarization (f). The relations and definitions of these parameters are illustrated below.

### 3.4. Permeate Flux

The permeate flux of the MD process is defined as the amount of produced pure water per unit time per unit area of the utilized membrane. Permeate flux could be expressed as follows [20,21,22]:

(6) $$\;m=C\left(P_{f}-P_{p}\right)$$

C, $P_{f}$, and $P_{p}$ are the mass transfer coefficient, vapor pressure at the feed side and vapor pressure at the permeate side, respectively.

### 3.5. Thermal Efficiency

The process thermal efficiency gives the ratio of the useful amount of heat that is utilized to evaporate the water to the total amount of heat supplied to the whole process. Thermal efficiency could be expressed as follows [12,20,23,24]:

(7) $$\;\eta =\frac{Q_{vap}}{Q_{vap}+Q_{conduction}}$$

$Q_{vap}$ and $Q_{conduction}$ are the heat utilized to evaporate the water in the feed channel and the heat transferred from the hot to cold side by conduction (lost heat), respectively.

### 3.6. Temperature Polarization Coefficient

The temperature polarization coefficient gives the amount of fluid/membrane interface temperatures’ deviation from the fluid bulk temperature. This affects the performance considerably, as the main driving force (vapor pressure) is a direct consequence of the membrane surface temperature. The temperature polarization coefficient could be expressed as follows [6,12,24,25]:

(8) $$f=\frac{T_{m/f}-T_{m/p}}{T_{b/f}-T_{b/p}}$$

$T_{m/f}$, and $T_{m/p}$ are the membrane surface temperatures at the feed and permeate side, respectively, while $T_{b/f}$, and $T_{b/p}$ are the bulk temperature at the feed and permeate channels, respectively.

### 3.7. Porosity

Finally, the porosity of the composite fabricated membranes could be obtained (after getting experimentally the mass of wet and dry samples) as follows [26]:

(9) $$\;\varepsilon =\;\frac{\left(m_{w}-m_{d}\right)/\rho_{i}}{\left(m_{w}-m_{d}\right)/\rho_{i}+m_{PS}/\rho_{PS}}$$

where $m_{w}$, $m_{d}$, and $m_{PS}$ are the wet mass, dry mass, and PS mass of the fabricated membrane, respectively; $\rho_{i}$ and $\rho_{PS}$ are the utilized alcohol density and PS density, respectively.

## 4. Numerical Solution

The three-dimensional (3D) model was constructed on Ansys 2020, which solves the controlling equations using the finite volume method. At first, the system was drawn, then discretized to be ready for solution. A mesh dependence test is required before performing the main simulation to obtain a solution that is not affected by the mesh size. In this regard, we used the software default meshing option to start with a coarse mesh and applied the system boundary conditions to obtain the solution. The system boundary conditions were velocity inlet (1.85–5.55 cm/s) at both feed and permeate inlet sections, and pressure outlet with zero gage pressure at both feed and permeate outlet sections, while the other walls were set to be adiabatic. The feed water temperature was 50–85 °C, while the permeate temperature was constant for the whole simulated case at 10 °C. The target solution is the permeate flux as it is one of the most important performance parameters. After that, the mesh was enhanced manually (increase the number of elements) and then the produced permeate flux was captured. The result of the mesh dependence test is listed in Table 1, which shows no further significant changes in the flux at case five, which records 0.1% relative error. Accordingly, case 5 with 400,000 elements was selected to be applied for all cases.

**Figure 1:**
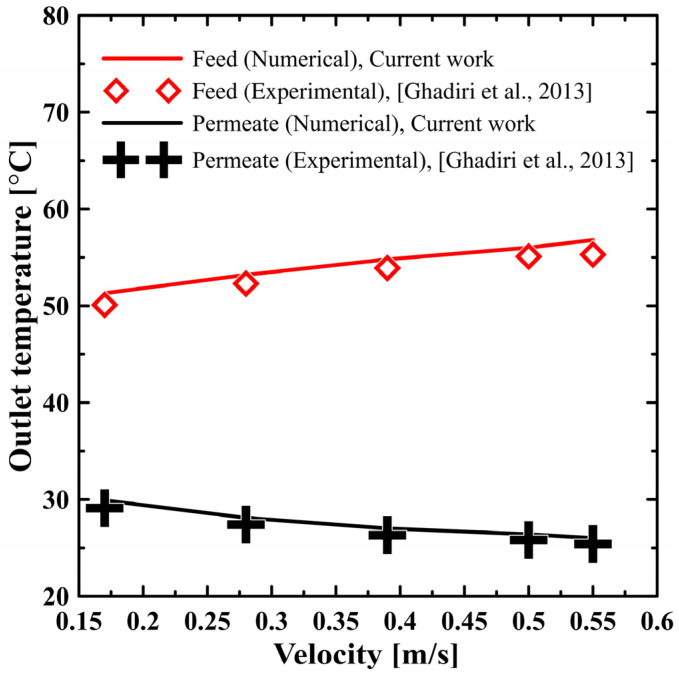
Model validation with Ghadiri et al. [27].

Furthermore, the introduced model has been experimentally validated with the available data [27]. The validation case captures the feed (hot salt water) and the permeate (cold pure water) outlet temperature at different flow velocities. This case was performed at typical conditions as mentioned in [27], which are 60 °C and 20 °C feed inlet and cold water inlet temperature, respectively; and salt concentration of 1% with a counterflow regime. Figure 1 presents the validation result, which excellently fits the experimental work with an error of only 2.8%. The model was modified to be a three dimensional model, validated with the same date and recorded with the same solution. Accordingly, this model could be simply modified to be used for any proposed future work.

The numerical model was solved under the following assumptions:

1. Laminar fully developed flow.
2. Temperature-independent properties for water.
3. The side walls are adiabatic.
4. Exclude any chemical reactions.

After that, the simulation will be started once the actual boundary conditions are applied to each boundary of the designed model to simulate the actual system and study the effect of target parameters on the system performance.

## 5. Result and Discussion

### 5.1. Characterization

**Figure 2:**
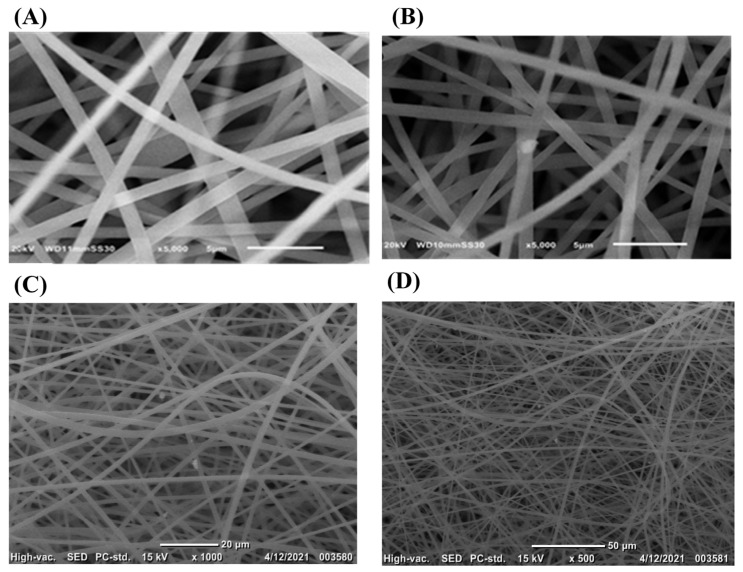
SEM image of (**A**) blank PS membrane and (**B**–**D**) composite PS/GNP membrane.

Figure 2 shows the SEM imaging for the PS and PS/GNPs fibrous membranes. As can be observed from Figure 2A the formed fibers are beadles, smooth, and uniform which confirm the suitability of the electrospinning conditions for the prepared polymer solution. The average fiber diameters were found to be 1.013 and 0.719 µm for the PS and PS/GNP, respectively. This decrease in fiber diameters may be attributed to the addition of the GNPs that enhanced the conductivity of the polymer solution [28].

**Figure 3:**
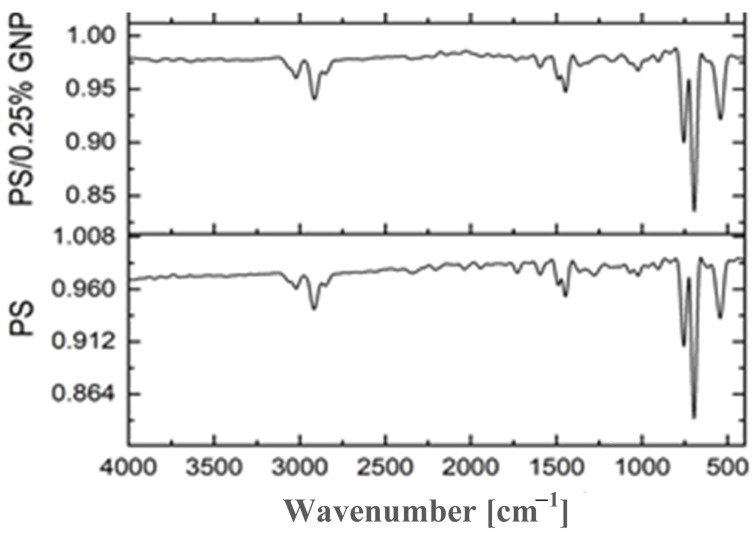
FTIR spectra of blank PS and composite PS/GNP membranes.

Figure 3 presents the IR spectra of the neat PS and composite PS/GNP membranes. The characteristic peaks observed at 3100–2800 cm^−1^ are attributed to the symmetric and asymmetric vibrations of C-H. The aromatic Mon substitution characteristic peaks are observed in the range of 2000–1680 cm^−1^. The pronounced peak at 1446.41 is assigned to the CH_2_ group bending vibration. The CH out-of-plane bending vibrations of the phenyl ring can be observed at 754.61 and 695.93 cm^−1^, while the out-of-plane deformation of the phenyl rings is observed at 543 cm^−1^. As the concentration of the GNPs increases, the reinforcement enhances. Hence, the PS characteristic peaks become stronger [5,29].

**Figure 4:**
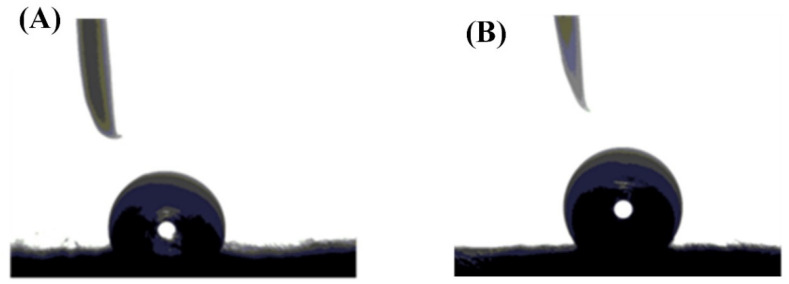
Contact angle measurement of (**A**) blank PS and (**B**) composite PS/GNP membranes.

The contact angle was measured and illustrated in Figure 4. the contact angle increased to become 91.68° because of adding 0.25 wt.% of GNP to the PS, compared to that of PS which was 73.79°. Finally, the porosities of the fabricated membranes were calculated using Equation (9), after obtaining the dry and wet masses of the fabricated membranes. The results show that the blank PS membrane recorded a porosity of 0.56, while that of composite PS/GNP was 0.8. Therefore, the porosity of the composite membrane was enhanced by approximately 44% due to the addition of GNPs.

### 5.2. Performance on DCMD

**Figure 5:**
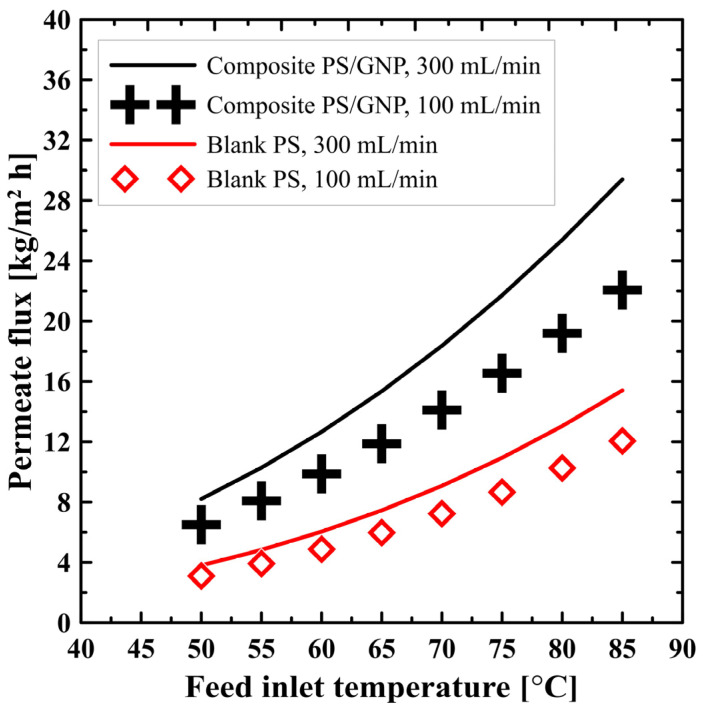
Effect of feed inlet temperature on the permeate flux at different flow rates for both blank PS and composite PS/GNP membrane.

Figure 5 shows the effect of feed inlet temperature on the MD productivity (permeate flux) at two different flow rates, for both the blank PS membrane and composite PS/GNP membrane. As can be seen from this figure, the productivity of PS/GNP membrane outperforms that of the blank PS membrane, as PS/GNP shows noticeable higher productivity. This higher performance could be ascribed to the composite membrane’s improved porosity due to GNP addition. For example, at 85 °C saltwater temperature and 300 mL/min flow rate, the productivity of the composite PS/GNP is 29.5 kg/m^2^ h, while that of the blank PS membrane is only 15 kg/m^2^ h, which is approximately double the amount. The impact of the flow rate on the productivity of the system is also clear from Figure 5. Raising the rate of flow was found to increase the system productivity. This could be explained as because increasing the flow rate improves the heat transfer in the feed channel, and the membrane surface temperature is nearly close to the fluid bulk temperature, which means low-temperature polarization (high-temperature polarization coefficient). For example, at the maximum studied saltwater temperature (85 °C), for the composite PS/GNP membrane, raising the rate of flow from 100 to 300 mL/min is found to increase the system productivity from 22 to 29.5 kg/m^2^ h, which matches with findings in [30,31]. Furthermore, Figure 5 also shows that feed inlet temperature affects the produced pure water amount significantly. As can be seen from the figure, increasing the saltwater temperature from 50 to 85 °C at a feed flow rate of 300 mL/min, for the composite PS/GNP membrane increases the productivity from 8.5 to 29.5 kg/m^2^ h, which is dramatically high. This is because the feed inlet temperature is the major motive of the driving force.

**Figure 6:**
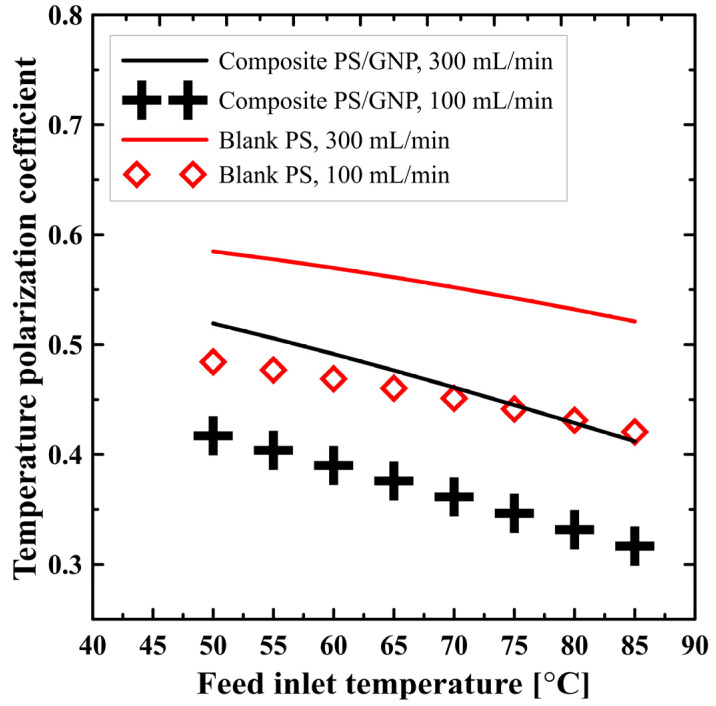
Effect of feed inlet temperature on temperature polarization coefficient at different flow rates for both blank PS and composite PS/GNP membrane.

Figure 6 shows the effect of operating parameters (flow rate and feed inlet temperature) on the temperature polarization coefficient for both the blank PS membrane and composite PS/GNP membrane. As a general view, increasing the feed inlet temperature decreases the temperature polarization coefficient. For example, at 300 mL/min flow rate for the composite PS/GNP membrane, increasing the hot water inlet temperature from 50 to 85 °C decreases the coefficient of temperature polarization from 0.52 to 0.41, which affects negatively the system performance. This could be attributed to the increased productivity at higher feed inlet temperatures, which utilizes much more heat to evaporate the water and acts as a heat sink at the membrane/feed channel interface. This deviates considerably the membrane/feed interface temperature far from the fluid bulk temperature, which increases the temperature polarization effect (decreases the temperature polarization coefficient). On the other hand, the flow rate has a positive effect on the temperature polarization coefficient, as can be seen from the figure. This could be explained, once again, because increasing the flow rate will improve the heat transfer coefficient and decrease the temperature polarization, which means an improvement in the temperature polarization coefficient. For example, raising the rate of flow from 100 to 300 mL/min at the maximum studied saltwater temperature, the composite PS/GNP shows an increase in the polarization coefficient from 0.31 to 0.41, which is a considerable amount. These results match well with those obtained by [32]. Moreover, the PS/GNP composite membrane has a lower temperature polarization coefficient than that of the PS membrane as could be seen from Figure 6. This could be justified as the improved porosity of the composite PS/GNP membrane decreases the mass transfer resistance and allows more heat to be utilized to evaporate water. This will act as a heat sink which results in a higher temperature polarization (lower temperature polarization coefficient) compared to that caused in the case of a blank PS membrane. At the greatest introduced rate of flow and saltwater temperature, blank PS recorded 0.52, while the composite PS/GNP membrane recorded 0.41.

**Figure 7:**
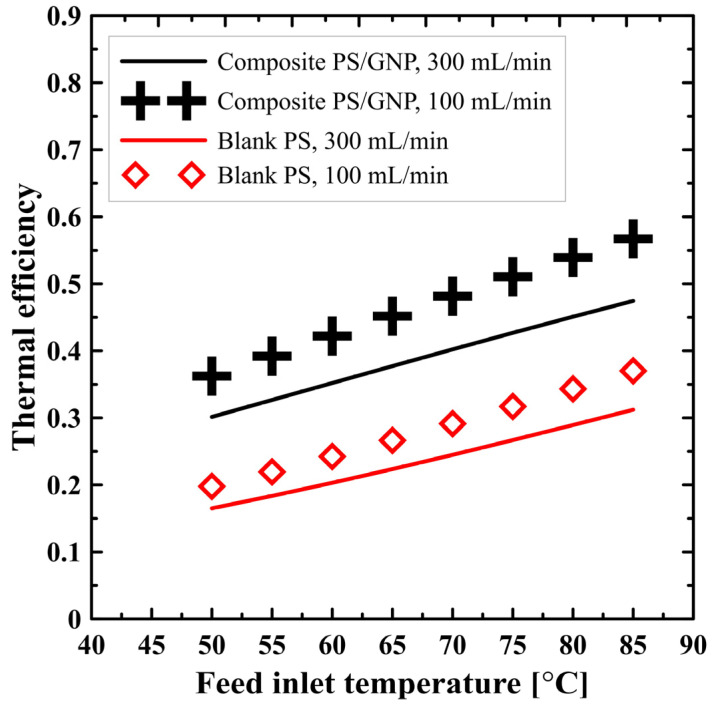
Effect of feed inlet temperature on the process thermal efficiency at different flow rates for both blank PS and composite PS/GNP membrane.

The effect of the feed inlet temperature and flow rate on the thermal efficiency of the DCMD process utilizing either blank PS membrane or composite PS/GNP membrane is shown in Figure 7. It can be seen that the process thermal efficiency increases because of increasing the feed inlet temperature. As illustrated in Figure 5, increasing the feed inlet temperature increases the productivity, and hence the heat utilized for evaporation increases, resulting in an improved thermal efficiency according to Equation (7). On the other hand, increasing the flow rate negatively affects the process thermal efficiency, because raising the rate of flow, while keeping the feed temperature fixed, requires adding much more heat. However, the increase in productivity is not comparable, and the overall trend of the thermal efficiency declines with the increasing flow rate. Overall, increasing flow rate increases the system productivity and decreases the system efficiency, so an optimization study is required to highlight the optimum operating flow rate after applying a spacer filled channel to enhance the efficiency at a higher flow rate. Additionally, noticeably, the composite PS/GNP membrane recorded higher efficiency than that of the blank PS membrane, as can be seen from Figure 7. This could be explained as a result of different porosity for each membrane type, since the porosity has a considerable impact on the whole performance, as illustrated in the previous sections. The higher porosity allows more flux to be transferred through the membrane, which utilizes much more heat. Therefore, the useful heat is considerably increased and improves the overall thermal efficiency.

**Figure 8:**
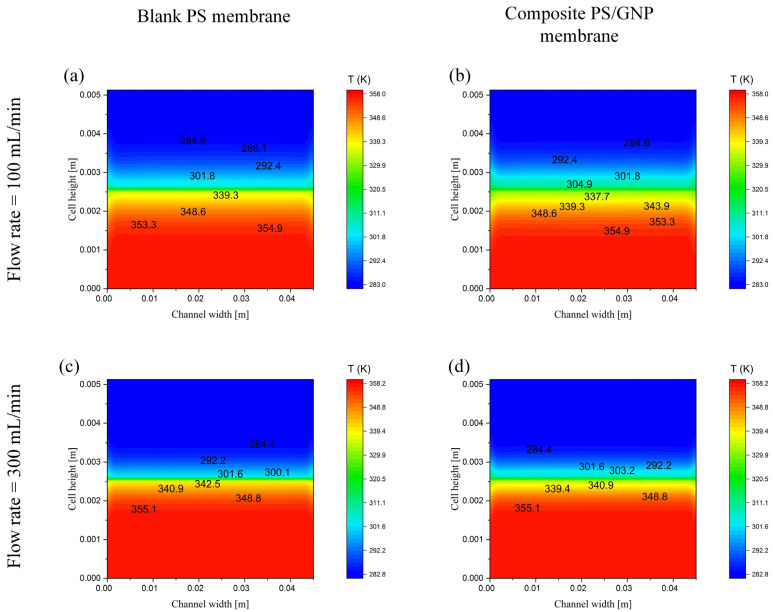
Contours at a section located half a way between inlet and outlet at 85 °C feed inlet temperature, (**a**) blank PS membrane at a flow rate of 100 mL/min (**b**) composite PS/GNP membrane at a flow rate of 100 mL/min (**c**) blank PS membrane at a flow rate of 300 mL/min and (**d**) composite PS/GNP membrane at a flow rate of 300 mL/min.

The temperature contours at maximum studied temperature, minimum and maximum introduced rates of flow for both fabricated membranes (blank PS and composite PS/GNP) are presented in Figure 8. Comparing the four introduced in Figure 8a–d, one can conclude that the highest thermal performance is that of the blank PS at the highest flow rate, as discussed in detail in the previous sections. These figures also show the temperature gradient from the hot side channel (feed channel) to the cold side (permeate channel), which follows the actual temperature gradient.

## 6. Conclusions

This current work introduces a fabrication of composite polystyrene with graphene nanoplates (PS/GNP) to produce a membrane suitable for the membrane distillation process and compared with the blank polystyrene (PS) membrane. The membranes were fabricated using the electrospinning technique and characterized using SEM, FTIR, contact angle, and porosity to introduce the validity of the fabrication processes. The fabricated composite membrane recorded an 80% porosity and contact angle of 91.68°, which proves the hydrophobic nature of the fabricated membrane. The performance of the fabricated membranes was studied on the direct contact membrane distillation (DCMD) model numerically. The introduced numerical model was validated with experimental work from the literature and showed excellent agreement. Furthermore, the performance of the fabricated membranes (blank and composite) was studied at different saltwater temperatures and different flow rates. The results show that the fabricated composite membrane has superior performance at different studied operating conditions. The composite PS/GNP membrane recorded permeate flux, temperature polarization coefficient, and thermal efficiency of 29.5 kg/m^2^ h, 0.41, and 0.475, respectively. Although the PS provided a good performance when used in the DCMD system, the composite PS/GNP membrane recorded considerably higher performance. Moreover, the results show that the hot water inlet temperature has a significant effect on the system productivity whatever the utilized membrane. In addition to that, operating the DCMD at a higher flow rate improves the overall system performance.

## Figures and Tables

**Table 1:** Mesh dependence study.

Case	Number of Elements	Permeate Flux [kg/m^2^ h]	% Error
1	50,000	12.30483	2.2
2	100,000	12.25667	1.8
3	200,000	12.20851	1.4
4	300,000	12.14831	0.9
5	400,000	12.052	0.1001
6	500,000	12.03995	Datum

## Data Availability

Not applicable.
